# From burnout to breakthrough: understanding the work-related stress-motivation Nexus in medical imaging professionals

**DOI:** 10.3389/fpubh.2024.1447853

**Published:** 2024-11-25

**Authors:** Alo Edin, Hikma Ali, Yenuse Molla, Miesa Gelchu, Ali Beyene, Angefa Ayele

**Affiliations:** ^1^Department of Epidemiology, School of Public Health, Institute of Health, Bule Hora University, Bule Hora, Ethiopia; ^2^Department of Radiology Technology College of Medicine and Health Science, Arbaminch University, Arba Minch, Ethiopia; ^3^Department of Medical Radiology Technology, College of Health Sciences, Addis Ababa University, Addis Ababa, Ethiopia; ^4^Department of Public Health, Institute of Health, Bule Hora University, Bule Hora, Ethiopia

**Keywords:** motivation, medical, imaging, professionals, work, stress

## Abstract

**Background:**

Work-related stress is a pervasive issue in the global occupational health landscape, ranking as the second-most common problem after low back pain. In Ethiopia, the healthcare sector is particularly affected by low staff motivation and stressed workers, which can significantly impact the functioning of health systems. However, when it comes to medical imaging professionals (MIPs), there is a lack of substantial evidence regarding the relationship between work-related stress and motivation. Therefore, this study aimed to address this gap by assessing the connection between work-related stress and motivation among medical imaging professionals in the study area.

**Materials and methods:**

To assess the relationship between work-related stress and motivation among medical imaging professionals in Addis Ababa, a facility-based cross-sectional study was conducted. The study included a sample of 420 medical imaging professionals, who were randomly selected using a simple random sampling technique. The data collected from the participants were entered into Epi Data 3.1 and analyzed using STATA 14.2. Bivariable and multivariable analyses were performed to explore the associations between work-related stress and motivation, taking into account various factors. To determine statistical significance, a *p*-value of less than 0.05 was used as the threshold.

**Results:**

This study found that 57.4% of the participants experienced workplace stress and 46.4% reported being motivated. We observed a negative relationship between work-related stress and job motivation. The findings indicated that sex (adjusted odds ratio (AOR) = 1.819, 95% confidence interval (CI): 1.125, 2.94), age (AOR = 0.186, 95% CI: 0.04, 0.78), radiation (AOR = 2.21, 95% CI: 1.377, 3.57), leadership (AOR = 2.54, 95% CI: 1.475, 4.385), financial incentives (AOR = 1.78, 95% CI: 1.052, 3.022), and profession category (AOR = 2.57, 95% CI: 1.006, 6.561) were significantly associated with workplace stress. In addition, profession category (AOR = 0.22, 95% CI: 0.78, 0.63), smoking (AOR = 0.262, 95% CI: 0.08, 0.77), permanent workplace (AOR = 4.321, 95% CI: 1.988, 9.39), monthly income (AOR = 4.589, 95% CI: 1.37, 15.288), and financial incentives (AOR = 3.39, CI: 2.093, 5.51) were significantly associated with job motivation.

**Conclusion:**

Based on the results of the current study, it can be inferred that work-related stress is substantial, whereas job motivation is lacking among medical imaging professionals. The study found that several factors, including sex, age, radiation, leadership, financial incentives, and profession category, were significantly associated with workplace stress. In addition, factors such as profession, smoking, permanent workplace, monthly income, and financial incentives were found to be significantly associated with job motivation. Notably, a negative relationship was observed between work-related stress and motivation.

## Introduction

According to the National Institute for Occupational Safety and Health (NIOSH), work-related stress is defined as “harmful physical and emotional responses that occur when the requirements of the job do not match the capabilities, resources, and needs of the worker” (1). Medical imaging professionals (MIPs) are classified into two types: diagnostic and therapeutic (2). Diagnostic radiographers work in the radiology department, including X-ray, ultrasound, computed tomography (CT), and magnetic resonance imaging (MRI) (3). They serve the majority of the hospital’s departments, including emergency, outpatients, operation theaters, and wards (4). In contrast, therapeutic radiographers work closely with doctors, nurses, physicists, and other oncology team members to treat cancer patients (5). Moreover, medical imaging professionals (MIPs) who are exposed to distress are brought to attention due to their role in guiding the technical preparation of both the instrumentation and the patient for the examination, setting the medical equipment for imaging, explaining the examination procedures to the patient, positioning the anatomic body region in the correct position for imaging, and preparing the dose of medicine to be injected for the exam (6, 7).

Stressful working conditions have also been shown to have a negative impact on job motivation (8). An individual’s level of willingness to exert and maintain effort toward organizational goals is referred to as worker motivation (9). Work-related stress is the second-most common occupational health issue in the world, after low back pain, which is the most prevalent occupational health problem (10). In this context, work-related stress is a rapidly growing global issue (11). For instance, a study on MIPs conducted in China and Australia found that the majority of MIPs—53.08% in China and 61.4% in Australia—experienced work-related stress (12). Furthermore, a study conducted by the radiology department of a public hospital in Gauteng, South Africa, reported that MIPs experience high levels of stress (13).

In Ethiopia, low staff motivation and work-related stress are factors that are crippling health systems and healthcare (14). Previous research has shown that Ethiopian health workers experience stress and lack motivation due to factors such as sex, age, education qualification, monthly salary, work experiences, educational opportunity, workload, leadership styles, work environment, and substance abuse (14–17). Studies conducted in Ethiopia showed that 46.9 to 78.3% of healthcare professionals had work-related stress (18, 19), while 63.63 to 19.5% were motivated at work (9, 14, 20). Work-related stress affects all healthcare professionals, but it is more prevalent among MIPs (19, 21). In Ethiopia, where there are few MIPs, there is a significant increase in workload, which predisposes individuals to increased work-related stress and, if not addressed, burnout (22). When MIPs are stressed, he or she may make mistakes when positioning the patient, aligning the intensity of the radiation, and writing the patient’s report (23). These kinds of mistakes can lead to inaccurate diagnoses, thereby affecting the quality of care and potentially jeopardizing patients’ lives (7).

Motivation can be either intrinsic or extrinsic (24), and it can lead to better performance and decreased levels of work-related stress among workers. A better understanding of health worker motivation is essential for designing effective healthcare delivery systems (25). However, despite the importance of understanding health worker motivation, there is relatively little evidence on this issue from low-and middle-income countries (26). Generally, previous studies on work-related stress and motivation have predominantly been conducted in developed countries (6, 27). Although their findings apply to Western populations, they may not be relevant to resource-constrained countries such as Ethiopia. Despite substantial studies on work-related stress and motivation among nurses and medical doctors, there is still very limited evidence documenting the relationship between work-related stress and motivation, particularly among medical imaging professionals (15, 28). Therefore, the current study aimed to assess the relationship between work-related stress and motivation among medical imaging professionals in the study area.

## Materials and methods

### Study settings

The study was conducted in Addis Ababa, the capital city of Ethiopia. In terms of healthcare facilities, Addis Ababa is equipped with a substantial number of hospitals and diagnostic centers. According to the Federal Ministry of Health’s (FMOH) 2020/2021 annual report, the city has 12 public hospitals, 24 private general hospitals, and 7 diagnostic centers (29). These facilities provide a diverse range of healthcare services to the population.

The study focused on medical imaging professionals in Addis Ababa, who play a crucial role in the healthcare system. The estimated number of medical imaging professionals in the city was 928, based on data obtained from the human resource offices of hospitals and the Ethiopian Radiographers and Radiologic Technologists Association (ERRTA).

The study was conducted for approximately 3 months, from 10 May to 29 July 2022. This timeframe allowed for sufficient data collection and analysis to explore the relationship between work-related stress and motivation among medical imaging professionals in Addis Ababa, Ethiopia.

### Study design

This was a facility-based cross-sectional study.

### Population

All randomly selected medical imaging professionals currently providing radiology services in Addis Ababa were included in the study.

### Sample size and sampling technique

The sample size was calculated using a single proportion formula with the following assumptions:


$$n=\frac{{\left(\frac{z\alpha }{2}\right)}^{2}p\;\left(1-p\right)}{d^{2}}$$

where *n* = minimum sample size required for the study,

*Z* = standard normal distribution (*Z* = 1.96) with a confidence interval (CI) of 95% and *α* = 0.05,

*P* = prevalence/ population proportion (*p* = 0.455), taken from a previous study (14), and *d* = margin of error (*d* = 0.05), with added 10% non-response. Finally, the largest sample size, 420, was taken as the final sample size.

**Figure 1:**
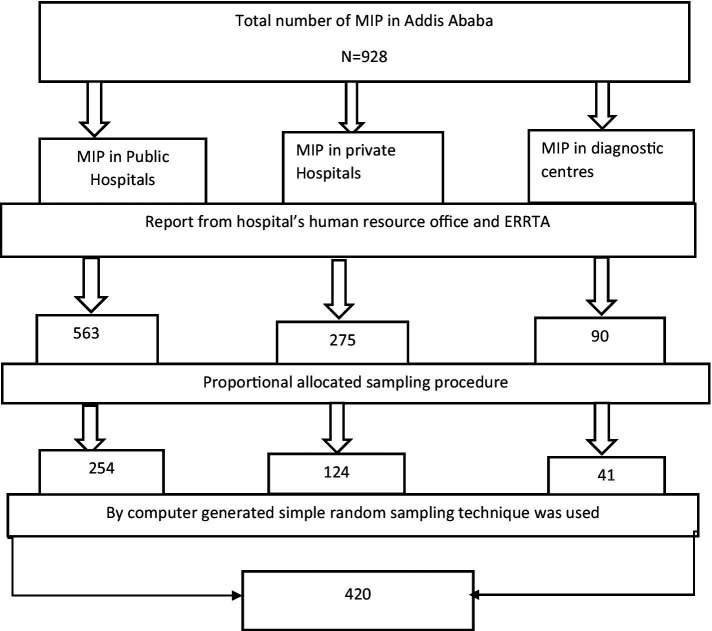
Schematic representation of the sampling procedure for the study on the relationship between work-related stress and motivation among medical imaging professionals in Addis Ababa, 2022.

To ensure a representative sample, a multi-stage sampling technique was used in this study. First, all diagnostic centers, public hospitals, and private hospitals in Addis Ababa were identified. Then, a comprehensive list of medical imaging professionals was obtained from both the ERRTA and the human resource offices of the hospitals and diagnostic centers. The sample size was determined based on this information. Next, the sample size was proportionally allocated to each hospital and diagnostic center to account for variations in size and capacity, ensuring that each site had a fair chance of being represented in the study. Finally, a simple random sampling technique was used to select study participants from each site. This method was used to minimize bias and ensure that each medical imaging professional had an equal opportunity to be included in the study (Figure 1).

### Data collection procedures and instruments

Data for this study were collected using a pretested and structured questionnaire, which was adapted from relevant literature that aligned with the study objectives (9, 14, 15). To ensure the reliability of the questionnaire, Cronbach’s alpha was calculated, yielding satisfactory results. The work-related stress items exhibited a reliability score of 0.84, organizational variables exhibited a reliability score of 0.88, personal factors exhibited a reliability score of 0.82, and job motivation exhibited a reliability score of 0.88.

The questionnaire consisted of four parts. Part I collected socio-demographic information through nine questions. Part II included six questions about organization-related variables. Part III included 13 items specifically designed to measure work-related stress among medical imaging professionals. These items were categorized into the following seven major subscales: workload, lack of support, conflict, uncertainty regarding patient treatment, dealing with death and dying, and organizational decisions. Each item was measured on a 4-point Likert scale, ranging from 1 to 4. A higher score indicated greater agreement with the statement, implying a higher level of perceived stress. The overall level of stress among the medical imaging professionals was determined by calculating the mean score of all 13 items. Part IV of the questionnaire assessed motivation using Likert scales with 16 questions, which were divided into six subscales.

The data collection process was carried out by two BSc nursing professionals, who were supervised by a health officer and the principal investigator. The data collection period was from 10 May 10 to 29 July 2022, and an electronic form (Google Form) was used. Prior to the actual data collection, permission was obtained, and a pretest was conducted at Ras Desta Damtew Memorial Hospital and Girum Hospital 1 week before the main study. Based on the pretest results, the questionnaire was modified to include the potential impact of radiation on work-related stress. After these preparations were completed, data collection commenced at all participating hospitals.

### Study variables

#### Dependent variables

The dependent variables were work-related stress and job motivation.

#### Independent variables

The independent variables included socio-demographic characteristics (age, sex, marital status, religion, educational status, profession category, years of experience, monthly income, and substance use), organizational factors (permanent place of work, workload, respect from other healthcare providers, responsibility for patient care, financial incentives, leadership, and radiation), and substance use (smoking, alcohol consumption, and khat chewing).

### Operational definitions

#### Work-related stress

The National Institute for Occupational Safety and Health (NIOSH) defines it as “the negative physical and emotional reactions that occur when the requirements of the job do not match the worker’s talents, resources, or needs” (30). It was measured by 13 items using a 4-point Likert scale and categorized dichotomously as stressed MIPs (if the participants scored ≥ the mean score) or not stressed MIPs (if the participants scored < the mean score) (15).

#### Job motivation

It is the desire or willingness to make an effort in one’s work (9). It was measured by 16 questions using a 5-point Likert scale and categorized dichotomously as motivated MIPs (if the participants scored ≥ the mean score) or unmotivated MIPs (if the participants scored < the mean score) (14).

#### Career development

It is defined as learning new skills and moving forward along one’s career path (31).

#### Financial incentives

MIPs can get from working institutions other than a monthly salary (14). It was categorized dichotomously as good financial incentive (if the participants scored ≥ the mean score) or not good financial incentive (if the participants scored < the mean score) (32).

#### Substance use

The use or consumption of any substance, such as alcohol, cigarettes, and khat, regardless of the amount and frequency of use over the past 3 months (33).

### Data quality control

To ensure the quality of the data, training was provided to the data collectors and supervisors regarding the aim, procedure, tool, consent form, how to approach the participants, and ethics prior to the data collection. The data collectors and supervisors had regular meetings every day to discuss challenges and develop a strategy for the following day. Before the data collection period started, a pretest study was conducted, which was not included in the study setting, to check for clarity, understandability, and simplicity of the items. The supervisors and investigator checked and reviewed all the formats to ensure completeness and consistency of the information collected, and immediate actions were taken accordingly. All collected data were evaluated for completeness and consistency in data management, storage, and analysis.

### Data processing and analysis

The data were entered into Epi Data 3.1 and then exported to STATA 14.2 for further analysis. Descriptive data were reported using frequency, percentage, mean, and median and were presented in the form of tables and texts. To examine multi-collinearity, the variance inflation factor was calculated, and it was determined that the maximum values for work-related stress and job motivation were 2.15 and 2.48, respectively, among all explanatory variables included in the multivariate logistic regression. The data were checked for outliers by generating a boxplot, and while there were outliers, they were not considered influential because the Cook’s distance was less than one.

To validate the overall model fit, omnibus coefficient tests and the Hosmer–Lemeshow tests were conducted. In addition, all assumptions of binary logistic regression were examined. Bivariate and multivariate analyses were performed using a binary logistic regression model at a 95% confidence level to determine the association between the dependent and independent variables. To identify the association, a *p*-value of ≤0.25 was used for inserting the variables into the multivariable analysis, and finally, the association was computed with a p-value of ≤0.05. An adjusted odds ratio (AOR) with a 95% CI and a p-value of ≤0.05 were used to determine the strengths associated with each outcome variable (work-related stress and motivation separately). Finally, Spearman’s rank correlation was conducted to assess the relationship between work-related stress and job motivation.

## Results

### Socio-demographic characteristics of the participants

Of the 420 potential participants in the study, 390 medical imaging professionals actively participated, resulting in a response rate of 93%. Among the participants, the majority were male, accounting for 233(59.7%) of the total. The age range of the participants spanned from 21 to 55 years, encompassing a diverse range of age groups. In terms of marital status, 207 (53.1%) reported being married. Regarding educational backgrounds, the majority of the respondents held a bachelor’s degree, comprising 272 (69.7%) of the participants. In addition, the majority of the participants, 266 (68.2%), identified as medical radiology technologists. In terms of work experience, the majority of the participants reported having 1–5 years of experience, accounting for 173 (44.4%) of the total. This suggests a relatively young and early career group of participants.

Finally, regarding monthly income, the majority of the respondents reported earning between 5,000 and 10,000 Ethiopian birr (ETB) per month, comprising 216 (55.4%) of the participants. This income range encompassed a significant portion of the participants’ monthly income (Table 1).

**Table 1:** Socio-demographic characteristics of the medical imaging professionals in Addis Ababa, Ethiopia, 2022.

Characteristics	Frequency	Percentage
Sex		
Female	157	40.3
Male	233	59.7
Age		
≤30	268	68.7
31–40	97	24.9
> 40	25	6.4
Marital status		
Single	161	41.3
Married	207	53.1
Widowed	7	1.8
Divorced	14	3.6
Separated	1	0.3
Educational status		
Diploma or Level IV	53	13.6
Bachelor’s degree	272	69.7
Master’s degree and above	65	16.7
Profession category		
Radiographer	54	13.8
Medical radiology technologist	266	68.2
MRT+ other fields	70	17.9
Work experience		
≤ 5	173	44.4
6–10	168	43.1
>11	49	12.6
Monthly income		
< 5,000 ETB	26	6.7
5,000–10,000 ETB	216	73.5
>10,000	148	37.9

### Substance use among medical imaging professionals

Based on the data presented in Table 2, a limited number of medical imaging professionals in Addis Ababa, Ethiopia, reported smoking cigarettes, consuming alcohol, and chewing khat. Specifically, only 27 (6.9%) reported smoking cigarettes, while 105(26.9%) admitted to consuming alcohol. Moreover, 59 (15.1%) reported chewing khat. These findings indicate that the majority of the medical imaging professionals in Addis Ababa do not use these substances (Table 2).

**Table 2:** Substance use among the medical imaging professionals in Addis Ababa, Ethiopia, 2022.

Characteristics	Frequency	Percentage
Smoking cigarette		
Yes	27	6.9
No	363	93.1
Consuming alcohol		
Yes	105	26.9
No	285	73.1
Chewing khat		
Yes	59	15.1
No	331	84.9

### Organizational characteristics of the participants

More than two-fifths (44.4%) of respondents were from public hospitals. According to 250 (64.1%) respondents, the daily average patient workload is high. The majority of the participants stated that other healthcare providers respect medical imaging professionals (317, 91.5%). More than half of the respondents (220, 56.4%) reported being responsible for patient care. A total of 190 (48.7%) participants perceived financial incentives for medical imaging professionals in the organization as inadequate, while 164 (42.1%) participants perceived leadership in the organization as fair. The majority of the respondents (233, 59.7%) perceived that working in radiation has an impact on workplace stress (Table 3).

**Table 3:** Organizational factors among the medical imaging professionals in Addis Ababa, Ethiopia, 2022.

Variables category		Frequency	Percentage
Permanent place of work	Public hospital	173	44.4
	Private hospital	124	31.8
	Diagnostic center	39	10.0
	Teaching hospital	54	13.8
Daily average patient workload	Low	8	2.1
	Medium	132	33.8
	High	250	64.1
Respected by other healthcare providers	Yes No	357 33	91.5 8.5
Responsible for patient care	Good Fair Bad	138 220 32	34.4 56.4 8.2
Benefits gained in the organization	Good	72	18.5
	Fair	128	32.8
	Bad	190	48.7
Leadership in the organization	Good	82	21.0
	Fair	164	42.1
	Bad	144	36.9
Working in the radiation area has an impact on work-related stress	Yes	233	59.7
	No	157	40.3

### Work-related stress among medical imaging professionals

**Figure 2:**
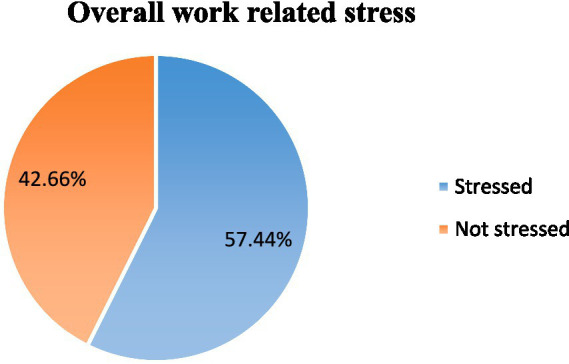
Overall prevalence of work-related stress among the medical imaging professionals in Addis Ababa, Ethiopia, 2022.

The study revealed that the overall level of work-related stress was found in 224 (57.4%) participants, who reported experiencing stress in their work. This indicates that the majority of these professionals are affected by work-related stress, underscoring the need for interventions and strategies to address and mitigate stress in the workplace (Figure 2).

Based on the mean score of each item related to work-related stress, the most stressful conditions reported among the medical imaging professionals, in descending order, were as follows: physician ordering an inappropriate imaging modality for a patient (2.97 ± 0.852), frequent changes in the unit of work (2.65 ± 0.963), insufficient staff to adequately cover the unit (2.61 ± 0.725), inadequate information from a physician regarding a patient’s medical condition (2.6 ± 0.817), watching a patient suffer (2.54 ± 0.828), and disagreement concerning the treatment of a patient (2.51 ± 0.838; Table 4).

**Table 4:** Work-related stress among medical imaging professionals in Addis Ababa, Ethiopia, 2022.

Variables	Never stressful		Sometimes stressful		Frequently stressful		Always/very frequently/stressful		Mean	Std. Dev
	*N*	%	*N*	%	*N*	%	*N*	%		
Not enough staff to adequately cover the unit	27	6.9	126	32.3	208	53.3	29	7.4	2.61	0.725
Not enough time to complete all of my tasks	43	11%	166	42.6	150	38.5	31	7.9	2.43	0.792
Not enough time to provide emotional support to the patient	47	12.1	158	40.5	148	37.9	37	9.5	2.45	0.825
Too many non-imaging tasks required, such as clerical work	95	24.4	124	31.8	138	35.4	33	8.5	2.28	0.927
Conflict with other health care providers	56	14.4	135	34.6	163	41.8	36	9.2	2.46	0.850
Disagreement concerning the treatment of a patient	41	10.5	157	40.3	145	37.2	47	12.1	2.51	0.838
Lack of opportunity to talk openly	57	14.6	150	38.5	150	38.5	33	8.5	2.41	0.840
Lack of support of my immediate supervisor	56	14.4	157	40.3	148	37.9	29	7.4	2.38	0.821
Inadequate information from a physician regarding the medical condition of a patient	32	8.2	142	36.4	165	42.3	51	13.1	2.60	0.817
A physician ordering inappropriate choice imaging modality	20	5.1	88	22.6	167	42.8	115	22.9	2.97	0.852
Feeling as my support is helpless in the case of a patient who fails to improve	44	11.3	168	43.1	143	36.7	35	9	2.43	0.808
Watching a patient suffer	41	10.5	140	35.9	165	42.3	44	11.3	2.54	0.828
Frequent change of unit of work	62	15.9	87	22.3	167	42.8	74	19	2.65	0.963

### Job motivation among medical imaging professionals

**Figure 3:**
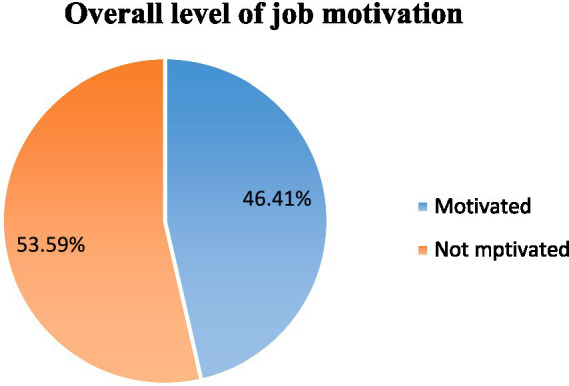
Overall prevalence of job motivation among the medical imaging professionals in Addis Ababa, Ethiopia, 2022.

The study revealed that the overall level of job motivation was found in 209 (53.6%) participants, who reported being motivated in their jobs (Figure 3).

The job motivation conditions reported among the medical imaging professionals, in descending order, were as follows: the organization offers good opportunities for continuing education (2.19 ± 1.283), sufficient service training to address skill gaps (2.35 ± 1.358), satisfaction with payment (2.47 ± 1.474), the organization recognizes hard work (2.81 ± 1.521), good employment benefits (2.81 ± 1. 455), satisfaction with management (2.82 ± 1.428), satisfaction with work (3.01 ± 1.476), motivation to work during daily activities (3.11 ± 1.485), and work providing personal achievement (3.29 ± 1.497) (SD) (Table 5).

**Table 5:** Job motivation among medical imaging professionals in Addis Ababa, Ethiopia, 2022.

Variables	Strongly Disagree		Disagree		Neutral		Agree		Strongly agree			
	*N*	%	*N*	%	*N*	%	*N*	%	*N*	%	Mean	SD
Motivated to work.	43	11	158	40.5	26	6.7	123	31.5	40	10.3	3.11	1.485
Satisfied with work	52	13.3	154	39.5	34	8.7	112	28.7	38	9.7	3.01	1.476
Work give personal achievement	31	7.9	155	39.7	23	5.9	148	37.9	33	8.5	3.29	1.497
Proud to work in this organization	36	9.2	134	34.4	29	7.4	137	35.1	54	13.8	3.31	1.471
Good employment benefit	56	14.4	185	47.4	29	7.4	103	26.4	17	4.4	2.81	1.455
Satisfied with payment	114	29.2	156	40	24	6.2	82	21	14	3.6	2.47	1.474
Organization recognized hard working.	76	19.5	158	40.5	28	7.2	108	27.7	20	5.1	2.81	1.521
Organization offers good opportunity to continue education	129	33.1	169	43.3	31	7.9	50	12.8	11	2.8	2.19	1.283
Satisfied with management.	48	12.3	190	48.7	38	9.7	103	26.4	11	2.8	2.82	1.428
Healthy working relationship	31	7.9	141	36.2	32	8.2	150	38.5	36	9.2	3.34	1.483
Equal treatment between colleagues.	26	6.7	137	35.1	33	8.5	156	40	38	9.7	3.41	1.466
Job and goal all are specific and clear	30	7.7	118	30.3	31	7.9	176	45.1	35	9	3.54	1.491
Organization has a clear objective what to achieve.	26	6.7	100	25.6	35	9	185	47.4	44	11.3	3.67	1.445
Sufficient service training to address skill gaps.	106	27.2	185	47.4	19	4.9	64	16.4	16	4.1	2.35	1.358
I prefer not to continue working in this organization.	42	10.8	84	21.5	49	12.6	140	35.9	75	19.2	3.48	1.432

### Factors Associated with work-related stress

The logistic regression analysis revealed several noteworthy findings. First, the female participants were found to be twice as likely to experience stress compared to the male participants (AOR = 1.819, 95% CI: 1.125, 2.94). In addition, participants aged 21–30 years were 81.4% less likely to experience work-related stress compared to those over the age of 40 years (AOR = 0.186, 95% CI: 0.044, 0.783). Medical imaging professionals working in radiation areas were found to be approximately twice as likely to experience work-related stress compared to their counterparts (AOR = 2.219, 95% CI: 1.377, 3.576). Participants working in organizations with poor leadership were twice as likely to experience work-related stress compared to those in organizations with good leadership (AOR = 2.54, 95% CI: 1.475, 4.385). Furthermore, the medical imaging professionals who reported having good financial incentives were approximately twice as likely to experience work-related stress compared to their counterparts (AOR = 1.783, 95% CI: 1.052, 3.022). The medical imaging participants who were not proficient in patient care responsibilities were twice as likely to experience work-related stress compared to their counterparts (AOR = 2.909, 95% CI: 1.79, 4.72). Finally, the radiographers were found to be approximately three times more likely to experience stress compared to their counterparts (AOR = 2.57, 95% CI: 1.006, 6.561; Table 6).

**Table 6:** Logistic regression analysis to identify factors associated with work-related stress among the medical imaging professionals in Addis Ababa, Ethiopia, 2022.

Variable	Category	COR (95% CI)	AOR (95% CI)	*p*- value
Sex	Female	1.687 (1.112–2.559)	1.819 (1.125–2.94)	0.015
	Male	1	1	
Age	21–30	0.384 (0.149–0.991)	0.186 (0.044–0.783)	0.022
	31–40	0.47 (0.172–1,281)	0.296 (0.083–1.062)	0.062
	>40	1	1	
Leadership	Good	1	1	
	Not good	3.54(2.249–5.59)	2.54 (1.475–4.385)	0.001
Working in radiation areas causes stress	Yes	1.026(1.329–3.087)	2.219 (1.377–3.576)	0.001
	No	1	1	
Financial incentives	Not good	0.273(0.158–0.473)	1.783 (1.052–3.022)	0.032
	Good	1	1	
Responsible for patient care	Good	1	1	
	Not good	3.205 (2.104–4.884)	2.909 (1.79–4.72)	0.0001
Profession category	Radiographer	3.025 (1.403–6.522)	2.57 (1.006–6.561)	0.048
	Medical radiology technologist	1.369 (0.808–2.321)	1.818 (0.936–3.531)	0.078
	MRT and other specializations	1	1	

### Factors Associated with job motivation

The results of the multivariable logistic regression analysis revealed some interesting findings. First, the radiographers were 83.4% less likely to have job motivation compared to the medical radiology technologists (AOR = 0.166, 95% CI: 0.06, 0.455). In addition, smokers were found to be 65.9% less likely to be motivated than non-smokers (AOR = 0.341, 95% CI: 0.12, 0.97).

Furthermore, participants working in diagnostic centers were approximately five times more likely to be motivated by their jobs compared to those working in public hospitals (AOR = 4.618, 95% CI: 1.729, 12.335). In terms of financial factors, participants earning higher monthly incomes were 56.7% more likely to have job motivation compared to their counterparts (AOR = 0.433, 95% CI: 0.254, 0.739). Moreover, the participants who reported having good financial incentives were 73.5% more likely to have job motivation compared to those who did not have such incentives (AOR = 0.265, 95% CI: 0.136, 0.514; Table 7).

**Table 7:** Logistic regression to identify factors associated with job motivation among the medical imaging professionals in Addis Ababa, Ethiopia, 2022.

Variable	Category	COR (95% CI)	AOR (95% CI)	*p*- value
Profession category	Radiographer	0.08 (0.032–0.197)	0.166 (0.06–0.455)	0.001
	Medical radiology technologist	0.406 (0.232–0.71)	0.572 (0.299–1.093)	0.091
	MRT+ other fields	1	1	
Cigarette smoking	Yes	0.380 (0.157–0.921)	0.341 (0.12–0.97)	0.044
	No	1	1	
Permanent place of work	Public hospital	1.155 (0.601–2.22)	1.605 (0.783–3.288)	0.196
	Private hospital	3.691 (1.869–7.28)	4.275 (2.018–9.053)	0.0001
	Diagnostic center	4.35 (1.807–10.46)	4.618 (1.729–12.335)	0.002
	Teaching hospital	1	1	
Monthly income	<5,000	0.118 (0.042–0.331)	0.259 (0.08–0.837)	0.024
	5,000–10,000	0.274 (0.176–0.426)	0.433 (0.254–0.739)	0.002
	>10,000	1	1	
Financial incentives	Good	0.185 (0.102–0.337)	0.265 (0.136–0.514)	0.0001
	Not good	1	1	

### Correlation between work-related stress and job motivation

The relationship between work-related stress and job motivation was assessed using Spearman’s correlation. The correlation for work-related stress was *R_s_* = 1.000, *p* < 0.002, whereas for job motivation, it was R_s_ = −0.166, *p* < 0.002. The current study reported that work-related stress negatively impacted job motivation (Table 8).

**Table 8:** Spearman’s rank correlation between work-related stress and job motivation among the medical imaging professionals in Addis Ababa, Ethiopia, 2022.

Spearman’s rho	Correlation Coefficient	P-value
Work-related stress	1.000	0.001
Job motivation	−0.166	0.001

## Discussion

Our study aimed to examine the relationship between work-related stress and job motivation among medical imaging professionals. The prevalence of work-related stress in our study was found to be 57.4%, which is lower than that reported in previous studies conducted in India (89.3% among MIPs (27)), Australia (61.4% among MIPs (34)), Gurage zone, Ethiopia (78.3% (19)), and Harar, Ethiopia (66.2% (33)). This discrepancy could be attributed to differences in measurement tools, sample sizes, and potential variations in the implementation of health service regulations and safety precautions in India and Australia.

In contrast, our study reported a higher prevalence of work-related stress than studies conducted among healthcare workers in Ghana (30.5% (35)), Mekelle, Ethiopia (46.9% (18)), Bahir Dar, Ethiopia (46.6% (16)), Arsi Zone, Ethiopia (53% (36)), medical imaging professionals in China (53.08% (12)), and Worabe, Ethiopia (56.3% (37)). The differences in prevalence may be due to variations in sample sizes and measurement tools used in these studies.

Regarding job motivation, our study found that 53.6% of the medical imaging professionals were unmotivated, while 46.4% were motivated in their jobs. This prevalence was lower than in previous studies conducted in West Amhara, Ethiopia (59% motivated (17)), Jima, Ethiopia (54.5% motivated (14)), and West Shoa, Ethiopia (63% motivated (9)). These differences may be attributed to variations in sample sizes, the populations studied, and the measurement scales used to assess job motivation.

Furthermore, our study reported a higher prevalence of job motivation than in several African countries, where 42.7% were motivated and 45.5% were unmotivated (14), as well as in the Gedo zone, Ethiopia (20). These discrepancies may be due to various factors influencing job motivation, which can vary from country to country, hospital to hospital, and even over time (28).

In this study, female participants were more likely to experience work-related stress than male participants. This finding is consistent with other studies conducted in Brazil, Worabe, Ethiopia, and Mekele, Ethiopia, which reported that female individuals experience higher work-related stress than male individuals (18, 37, 38). In contrast to this study, studies conducted in Jordan and India reported that male individuals were more stressed than female individuals (27, 39). However, the studies conducted in Jimma, Ethiopia, Ghana, and Iran did not find any statistically significant differences in perceived work-related stress based on sex (15, 35). The differences in results may be due to varying role expectations for male and female individuals across the globe, particularly in male-dominated societies such as Ethiopia (37).

.The current study revealed that participants aged 21–30 experienced less stress than those in the >40 years age group. This finding is consistent with a study conducted in China, which showed that older medical imaging professionals experienced more work-related stress. This was explained by a decline in tolerance and enthusiasm for work in demanding situations as age advances (12). Contrary to this finding, a study conducted in Worabe, Ethiopia, showed that the younger age group may have less experience and fewer coping skills, thereby making them more vulnerable to stress in the workplace (37). However, studies conducted in Germany, Jimma, and Gondar found no statistical significance between age and work-related stress (15, 40, 41). Age-related variations in emotional and physiological responses to stress have been observed, despite discrepancies in findings across individual studies (41).

This study revealed that participants working in radiation areas were twice as likely to experience work-related stress compared to their counterparts. This finding is consistent with the findings from other studies conducted in Iran, where a radiation work environment was statistically significantly associated with work-related stress (42). Similar findings were reported in studies conducted in China and India (12, 27).

The current study found that stress levels were higher among participants in organizations with poor leadership than those with fair leadership. Similarly, studies conducted in Japan and Indonesia reported that work-related stress was statistically significantly associated with leadership, especially transformational leadership (43, 44). A study conducted in the U.S. reported that leader mindfulness was an important interpersonal factor in reducing hindrance-related work stress (44). This difference may be due to variations in sample sizes, methods used for data collection and analysis, and sample representation.

This study found that participants who did not receive good financial incentives were more likely to experience work-related stress compared to those who did receive good financial incentives. This finding is consistent with a study conducted in Worabe, which indicated that financial incentives were a statistically significant factor affecting work-related stress (37). However, studies conducted in Jimma and Bahir Dar found no statistically significant difference between financial incentives and work-related stress among healthcare workers (15, 45). This difference may be due to the study setting.

The current study revealed that radiographers were twice as stressed as medical radiology technologists, and there was a significant association between profession category and work-related stress. The findings of previous studies showed that more educated healthcare workers have higher expectations regarding the extrinsic aspects of their jobs and are therefore more dissatisfied if these expectations are not met (16, 27). A study conducted in Mekelle found no statistically significant association between profession category and work-related stress (18).This discrepancy may be due to two reasons: first, a lack of clarity in their job description regarding the MIP, and second, a possible explanation being differences in workforce/staff allocation across units.

The participants who were not proficient in patient care responsibilities were more likely to experience work-related stress than those who were responsible for patient care. This is consistent with previous studies conducted in Iran and Australia, which indicated that individuals with a high level of patient care responsibilities are physically and psychologically better than those without, and their stress levels are lower (46, 47). The difference may be due to socio-cultural variations between countries. Improved healthcare delivery across the country could also be another factor contributing to this discrepancy.

This study found that the radiographers were less likely to have job motivation compared to the medical radiology technologists. This finding is supported by studies conducted in Central Ethiopia and West Amhara, which demonstrated that healthcare professionals with higher education levels had the highest mean motivation scores (9, 17). Similarly, a cross-sectional study conducted in Jimma, Ethiopia, found that job motivation was significantly associated with professional category (14).

The findings of this study suggested that smokers were less likely to be motivated than non-smokers. This aligns with a study conducted in the U.S., which also found a significant association between smoking and decreased motivation (48). Similarly, a study conducted in Germany found that smoking does not help people relax but rather decreases motivation (49). These consistent findings across different studies and settings indicate that there may be a negative impact of smoking on motivation. However, it is important to consider that socio-cultural differences between countries may contribute to discrepancies in the findings. Attitudes toward smoking and its effects on motivation may vary across different cultures and societies.

The finding from this study suggested that the MIPs working in diagnostic centers were more likely to be motivated by their jobs than those working in public hospitals. Similarly, an Egyptian study found that staff in the private sector were more motivated than those in the public sector (50). A study conducted in Ethiopia found that the majority of public-sector healthcare providers were unmotivated (51). These discrepancies could be attributed to differences in sample sizes and staffing/workload, all of which can influence motivation levels in different contexts.

The current study found that participants with higher monthly incomes were more likely to have job motivation than those with lower monthly incomes. This finding is consistent with studies conducted in Egypt, Ethiopia, and Gedio (20, 50, 51). These consistent findings across different studies and settings suggest that income level is an important factor to consider when assessing job motivation. Higher incomes can serve as a tangible reward that acknowledges and values the efforts and contributions of individuals, thereby enhancing their motivation to perform well in their jobs.

This study found that the MIPs who have good financial incentives were more likely to have job motivation compared to those who did not have access to financial incentives. This finding is consistent with studies conducted in Nigeria (52) and Gedeo, Ethiopia, which found that financial incentives were the most important motivating factor in improving performance (20). Moreover, healthcare professionals earning monthly financial benefits had higher motivation scores than healthcare professionals who did not receive any financial benefits (9).

This discrepancy could be attributed to differences in study participants and their specific contexts. It is crucial to consider these variations in study samples and settings when interpreting the results and generalizing the findings. Although financial incentives may have a significant impact on job motivation for some healthcare professionals, they are not a universal factor and may vary depending on individual circumstances and organizational contexts.

The current study revealed that stress has a negative impact on job motivation. This finding is in line with a study conducted in Uganda, which revealed that job motivation has long been thought to be influenced by work-related stress (25). A study conducted in China found that work-related stress has a negative impact on job motivation (8). Moreover, work-related stress and job motivation among healthcare workers are important determinants of their performance and, ultimately, the quality of care they provide (53). Work-related stress plays a significant role in decreasing work motivation and increasing motivation to leave the job (9). Similarly, work-related stress is significantly positively associated with work motivation (8, 53, 54). It is important to note that the discrepancies in the findings of different studies could be attributed to variations in the tools used to measure work-related stress and motivation, as well as differences in study settings. These variations can influence the interpretation and generalizability of the results. Therefore, it is crucial to consider these factors when comparing and analyzing the findings of different studies.

### Strength and limitations of the study

The primary strength of this study was its incorporation of essential assessments of work-related stress and job motivation among medical imaging professionals, which had not been studied well by other researchers, particularly within this study population. However, the cross-sectional nature of this study precluded a clear identification of the cause-and-effect relationship between the independent and dependent variables. In addition, the study might have been subjected to self-response bias.

## Conclusion

This study revealed that work-related stress was remarkably high, while job motivation was notably low among the medical imaging professionals. Furthermore, a negative correlation was detected between these two variables. Work-related stress was linked to various factors such as sex, age, radiation exposure, leadership, financial incentives, responsibility for patient care, and professional category. On the other hand, job motivation was associated with factors such as professional category, smoking habits, permanent workplace, monthly income, and financial incentives. The study suggests that work-related stress and job motivation should be incorporated into health policies and guidelines. Future research is recommended to use qualitative methods to uncover additional factors that could potentially influence work-related stress and job motivation among medical imaging professionals.

## Data Availability

The original contributions presented in the study are included in the article/supplementary material, further inquiries can be directed to the corresponding author.
